# Delivering Clinically on Our Knowledge of Oxytocin and Sensory Stimulation: The Potential of Infant Carrying in Primary Prevention

**DOI:** 10.3389/fpsyg.2020.590051

**Published:** 2021-04-29

**Authors:** Henrik Norholt

**Affiliations:** SomAffect - The Somatosensory & Affective Neuroscience Group, Liverpool, United Kingdom

**Keywords:** oxytocin, skin-to-skin contact, infant carrying, intervention, prevention, affective touch, caregiving

## Abstract

Oxytocin (OT) is one of the most intensively researched neuropeptides during the three past decades. In benign social contexts, OT exerts a range of desirable socioemotional, stress-reducing, and immunoregulatory effects in mammals and humans and influences mammalian parenting. Consequentially, research in potential pharmacological applications of OT toward human social deficits/disorders and physical illness has increased substantially. Regrettably, the results from the administration of exogenous OT are still relatively inconclusive. Research in rodent maternal developmental programming has demonstrated the susceptibility of offspring endogenous OT systems to maternal somatosensory stimulation, with consequences for behavioral, epigenetic, cognitive, and neurological outcomes. A translation of this animal research into practically feasible human parenting recommendations has yet to happen, despite the significant prevention potential implied by the maternal developmental programming research. Extended physical contact with full-term healthy infants in the months following birth (infant carrying) might constitute the human equivalent of those specific rodent maternal behaviors, found to positively influence emerging OT systems. Findings from both OT and maternal programming research parallel those found for infants exposed to such extended parental physical contact, whether through skin-to-skin contact or infant carrying. Clinical support of parents to engage in extended physical contact represents a feasible intervention to create optimum conditions for the development of infant OT systems, with potential beneficial long-term health effects.

## Highlights

- Oxytocin is implicated in positive physiological and psychological adaptations, with significant consequences for lifelong health.
- Oxytocin systems are shaped by early bidirectional parent–infant somatosensory stimulation.
- In oxytocin research, concluding remarks frequently highlight the vital necessity to translate findings to parenting practices.
- No medical guidelines exist to support optimum early development of oxytocin systems in humans.
- Increasing clinical support for parental full-term infant carrying/skin-to-skin contact constitutes a promising avenue to influence oxytocin systems.

## Introduction

In the past decades, two related research domains have been the subject of intense exploration: the neuropeptide oxytocin (OT) and rodent maternal behavioral programming of offspring neurological and behavioral development (Meaney, 2001; Buschdorf and Meaney, 2015; Insel, 2016). OT is implicated in the regulation of maternal caregiving as well as socioemotional and physiological development and health (Pedersen and Boccia, 2002; Clodi et al., 2008; Insel, 2010; Rilling and Young, 2014; Kim and Strathearn, 2016; Carter, 2017; Jones et al., 2017; Ding et al., 2019; Strathearn et al., 2019; Buemann and Uvnäs-Moberg, 2020).

In healthy human subjects, employing intranasal OT administration have shown anxiolytic effects (Heinrichs et al., 2003), enhanced recognition of emotional facial expressions (Kirsch et al., 2005; Bartz et al., 2010; Hurlemann et al., 2010; Marsh et al., 2010), increased interpersonal trust, especially among in-group members (Kosfeld et al., 2005; De Dreu et al., 2010), and elevated empathic concern for conspecifics (MacDonald and MacDonald, 2010).

These findings have sparked considerable research interest in applying OT administration to reduce or ameliorate mental disorders, including autism (Meyer-Lindenberg et al., 2011; Geng et al., 2018), depression (MacDonald et al., 2013; Kim et al., 2014), anxiety (Jones et al., 2017), social phobia, schizophrenia (Jarskog et al., 2017), insecure attachment (Buchheim et al., 2009), and addiction (Pedersen, 2017). At this stage, the most robust positive outcomes have been demonstrated for alcohol addiction (Pedersen et al., 2013; Pedersen, 2017) and autism (Hollander et al., 2003, 2007; Guastella et al., 2010; Tauber et al., 2011; Aoki et al., 2014; Watanabe et al., 2015; Yamasue, 2016; Higashida et al., 2019).

High doses of OT administration (80 IU/day) over several weeks have significant positive effects on schizophrenia (Zheng et al., 2019). Similarly, repeated intranasal oxytocin administration early posttrauma reduces subsequent PTSD symptom development in recently trauma-exposed emergency department patients with high acute PTSD symptoms (Frijling, 2017).

In non-clinical mothers, OT infusion during labor or following planned cesarian induced positive personality adaptations (Jonas et al., 2008; Velandia, 2012).

Relevant to the scope of the present article—primary prevention across both psychological and physiological domains—OT administration has been shown to induce weigth loss in humans and improve control of energy intake (Lawson et al., 2020) and may also offer potential for improved cardiovascular health through improved immune system functioning (Buemann and Uvnäs-Moberg, 2020).

For some domains, such as postpartum maternal depression and its interaction with the quality of parental caregiving, current effects of OT administration are equivocal (Scantamburlo et al., 2015; Mah, 2016; De Cagna et al., 2019).

The ambiguous outcomes of OT administration in relation to some of social disorders are in part ascribed to an enhanced “social salience effect” whereby OT increases the salience of both positive or negative social stimuli, resulting in increased prosocial behavior in positive environments, but producing defensive and, ostensibly, “antisocial behavior” in negative (competitive or potentially threatening) environments, which, however, when the behavior is viewed contextually, represents an adaptive response to social unsafe or provoking cues (Bartz et al., 2011; Bakermans-Kranenburg et al., 2012; Bakermans-Kranenburg et al., 2013; Tabak, 2013; Ne’eman et al., 2016). Although OT has been mostly linked to prosocial behavior, it also acts a “boundary setting” hormone, influencing, e.g., maternal aggression toward intruders, experimentally placed in the maternal cage with the pups present (Bosch, 2013). For example, among high-anxiety rodent dams, the levels of intracerebral release of OT correlates with maternal aggression toward a virgin intruder (Bosch et al., 2005), and in low-anxiety strains, synthetic OT infusion in the paraventricular nucleus through retrodialysis increases maternal intruder-directed aggression (Bosch and Neumann, 2012). Attachment status/caregiving history and genetic composition is also implicated in differential outcomes of OT administration (Macdonald, 2012; Riem et al., 2013; Feldman and Bakermans-Kranenburg, 2017). Much of the OT administration research has been based on single-dose treatments, whereas many of the conditions attempted to be treated are considered to be of a permanent or long-lasting nature, necessitating long-term treatments—for a review of chronic OT administration, see Horta et al. (2020). Furthermore, as evidenced by animal research, the differential effects of OT in different species is partially mediated by differential species-specific distributions of OT receptors (Insel, 2010). Exogenously administered OT reaches very different (peripheral) OT receptor sites, compared to endogenously released OT (activated through, e.g., somatosensory stimulation), in part because exogenously administered OT may not readily cross the blood–brain barrier (Leng and Ludwig, 2016; Uvnäs Moberg et al., 2019). Additionally, more work is required to establish relevant dosages of OT (Insel, 2016; Leng and Ludwig, 2016).

However, there are other pathways than pharmacological remedies for the stimulation of oxytocinergic mechanisms. Several veteran OT researchers have highlighted the early life period as one of great developmental plasticity for OT systems, calling for the translation of OT animal research to early human interventions, but regrettably, so far, there has been few concrete proposals regarding the potential nature of such an intervention (Francis et al., 1999; Pedersen and Boccia, 2002; Pedersen, 2004; Carter et al., 2009; Hammock, 2015), with Champagne (2008) and Feldman et al. (2014) as notable exceptions.

One of the salient intersections of research in OT systems functioning and rodent maternal programming, respectively, is the effects of non-noxious tactile stimulation (Francis et al., 2000; Uvnäs-Moberg et al., 2014), the latter requiring no medical involvement. The purpose of this article is to investigate the potential translation of the combined OT-related tactile stimulation and maternal programming research into primary prevention. Primary prevention aims to prevent disease before it occurs by altering behaviors that can lead to disease or injury and increasing resistance to disease or trauma/injury should exposure occur (Institute for Health & Work, 2015). Given the premise that suboptimum or dysfunctional OT functioning is implicated in a range of mental and physical illnesses (Strathearn, 2011; McQuaid et al., 2014; Kim, 2015; Wang et al., 2015; Colonnello et al., 2017), the essential prevention question becomes whether there may be ways to universally support an optimum development of the OT system.

One potential candidate for such primary intervention involves the practice of clinically recommended and supported extended parent–infant physical contact in the first months (EPIC), following birth at term (Norholt, 2020). Such extended parent–infant physical contact can be obtained through skin-to-skin ventral–ventral contact (Bystrova et al., 2003, 2009; Bigelow and Power, 2012) or through infant carrying with both caregiver and infant clothed, whether ventral–ventral (colloquially known in Western cultures as “babywearing”) (Williams and Turner, 2020), lateral–ventral (“hip-carrying”), or dorsal–ventral (“back-carrying”) (Graham et al., 2015).

It would appear obvious to include in the argumentation for such an intervention the full range of studies, which have sought to correlate plasma or saliva levels of OT to human behavioral phenotypes, especially in relation to both maternal and paternal caregiving. However, it is not clear at this stage whether such peripherally derived measures reliably reflect central nervous system concentrations of OT (Altemus et al., 2004; Kagerbauer et al., 2013; Carson et al., 2015). The interpretation and comparability between some of the human studies are also hampered by concerns regarding the validity of widely used commercially available immunoassays, which quantify peripheral OT concentrations in plasma and saliva (Horvat-Gordon et al., 2005; Szeto et al., 2011; McCullough et al., 2013; MacLean et al., 2019). One complication when comparing measurements of plasma and saliva OT levels arises from the finding that saliva OT appears to be a weak correlate for plasma OT (Quintana et al., 2018).

Furthermore, the two dominant approaches to measuring OT in plasma, radioimmunoassay and enzyme immunoassay, result in OT levels that are 100-fold different and, even more bewildering, are not correlated (Szeto et al., 2011; McCullough et al., 2013). Radioimmunoassays detect the intact OT hormone, whereas enzyme immunoassays may have less specific binding properties, which consequently detect both OT fragments as well as the principal OT hormone, leading to higher OT values (McCullough et al., 2013; Uvnäs Moberg et al., 2019). Our understanding and measurement of the impact of OT on physiological and behavioral systems are furthermore complicated by the possibility that oxytocin may be a principial hormone, exerting its effects in part through larger precursor OT molecules or converted active OT fragments with more specific effect profiles, the latter potentially binding to other receptors (such as alpha-2-adrenoreceptors) than the classical OTR (Uvnäs Moberg et al., 2019). For the most recent insights into validation of OT measurements, please refer to MacLean et al. (2019), but consider also Uvnäs Moberg et al. (2019) work on OT fragments. For reviews of OT in human parenting (e.g., Feldman, 2016; Feldman and Bakermans-Kranenburg, 2017).

Thus, the article will provide a brief summary of relevant OT research, with an emphasis of the effects of non-noxious tactile stimulation as well as the rodent maternal developmental programming research, which is centered on variations in maternal tactile stimulation—pup-directed licking and grooming and maternal skin-to-skin contact. The modalities and effects of extended parent–infant contact are described and discussed in relation to OT sensory stimulation and maternal developmental programming research. Clinical and policy implications are elaborated.

## General Aspects of OT

OT and the closely related peptide hormone vasopression (VP) are among the oldest mammalian hormones and are considered to have been central to human evolution (Robinson et al., 2019). Here, the description is restricted to OT (see, e.g., Carter, 2017 for a description of OT and VP dynamics). OT dynamically modulates the autonomic nervous system, affects vagal and immune system functioning, and exert pervasive adaptive functions on social behavior, which again predicts both emotional and physical health (Puig et al., 2013; Carter, 2014).

OT is a neuropeptide consisting of nine amino acids, produced in two nuclei within the hypothalamus, i.e., the paraventricular nucleus and the supraoptic nucleus. Within these two cell groups, OT is produced in two types of cells: the magnocellular neurons and the parvocellular neurons. OT has a dual function as both a hormone and as a neurotransmitter. Its hormonal effects are exerted via magnocellular neurons within the paraventricular nucleus and the supraoptic nucleus, which project to the neurohypophysis wherefrom oxytocin is released into the circulation, where it mediates the classically known OT effects on uterine contraction during labor and milk ejection during breastfeeding. However, the magnocellular neurons are furthermore provided with axon collaterals, which reach other regulatory parts of the brain, including the frontal cortex, the amygdala, and the anterior pituitary, where OT acts as a neurotransmitter. The parvocellular OT neurons emanating from the paraventricular nucleus also contribute to the neurotransmitter function of OT through their projections to many different cortical, limbic, and brainstem areas of the brain (Uvnäs-Moberg, 2015). Under conditions of intense stimulation of OT secretion, where OT is released not only from the axons of OT-producing neurons but also from the cell body and dendrites, OT can also exert its effects through diffusion to nearby and even distant parts of the brain to which no OT neurons project but which contain OT receptors (Ludwig and Leng, 2006). Such intense stimulation may furthermore induce temporary morphological changes in the OT-producing areas in the hypothalamus, causing surrounding supporting glia cells to withdraw, inducing functional changes in that the OT-producing cells gradually synchronize their electrical activity, leading to their releasing of electrical bursts, which induce pulses of OT into the circulation, as seen during labor and breastfeeding (Hatton et al., 1984; Theodosis, 2002).

### Oxytocin Production in Extrahypothalamus Sites

Production of OT is not restricted to the hypothalamus but has been found to also occur in the uterus, ovaries, amniotic fluid, placenta, heart, blood vessels, gastrointestinal tract, testes, kidneys, and thymus, where they exert local (paracrine) effects (Gimpl and Fahrenholz, 2001).

### OT Receptors

OT exerts its functions by binding to identical OT receptors (OTRs), present in both the brain and in the peripheral organs (uterus, mammary glands, kidneys, stomach, heart, blood vessels). Blocking OT receptors of the uterine kind with antagonists do not nullify all effects induced by OT, so additional as yet unidentified OT receptors may also exist. Furthermore, OT elongated preoxytocin molecules (Amico and Hempel, 1990; Green et al., 2001) as well as degradation products can also produce specific effects through binding to such unidentified OT receptors or by binding to opioid receptors and alpha-2-adrenoreceptors (Uvnäs-Moberg, 2015; Uvnäs Moberg et al., 2019).

### Genetic and Epigenetic Factors

The OT network, consisting of both OTergic neurons and OTR, is capable of undergoing dynamic changes, not least in the early stages of life, in an intricate interplay of the caregiving environment and genetic susceptibility to the caregiving environment (Toepfer et al., 2017).

## Non-Noxious Tactile Stimulation and OT in Rodents and the Implication of the Research for Humans

### OT Release in Response to Non-noxious Somatosensory Stimulation

Some of the earliest indicators of oxytocin release in response to stimulation of sensory nerves originating in the skin spring from rodent research, conducted by the oxytocin research pioneer, Kerstin Uvnas-Moberg. When anesthetized adult rats were exposed to electric stimulation of the sciatic or vagal nerves or received gentle stroking on their backs, plasma OT levels rose by 30–184% or 181%, respectively (Stock and Uvnäs-Moberg, 1988). Employing either vibration, warm temperature, or electroacupuncture to male anesthetized rats similarly increased plasma and cerebrospinal fluid OT levels (Uvnäs-Moberg et al., 1993). In humans, massage-like hand movements applied by newborn babies to their mother’s breast increased maternal plasma oxytocin levels (Matthiesen et al., 2001). Similarly, 15 min of moderate-pressure Swedish massage significantly increased plasma oxytocin levels in both men and women (Morhenn et al., 2012).

### Effects of Non-noxious Sensory Stimulation

Non-noxious sensory stimulation directed toward somatosensory nerves in either conscious or unconscious rodents acts through differentiated brain pathways to, respectively, reduce sympathetic nervous system activity as well as to increase parasympathetic nervous system activity. These physiological changes give rise to a range of effects, such as increased functioning of the gastrointestinal tract (Holst et al., 2005), increased sociability (Wei et al., 2013), increased pain threshold (Agren et al., 1995), as well as significant antistress effects (decrease in blood pressure and cortisol levels) (Araki et al., 1984; Lund et al., 1999; Wei et al., 2013). Interestingly, the effects of intercerebroventricular OT administration (which bypasses the blood–brain barrier) greatly resembles the effects induced by non-noxious sensory stimulation (Uvnäs-Moberg, 2015). Considering the abovementioned findings that OT is released in response to such stimulation, it is likely that OT is involved in mediating the effects described for non-noxious sensory stimulation. That OT mechanisms are involved is supported by the finding that the elevated pain threshold induced by ventral stroking in rodents is thwarted by the administration of an OT antagonist prior to the tactile stimulation (Uvnäs-Moberg et al., 1993; Agren et al., 1995).

In sum, non-noxious somatosensory stimulation in rodents and humans and its resultant contextually adapted OT response appear to reduce sympathetic nervous system activity as well as activate the parasympathetic nervous system, enabling a state of growth and relaxation or of calm and connectedness, a state that is optimum for both a growing infant and for its caregiving parents, combining the twin goals of supporting infant physical growth/state organization as well as the development a mutual emotional connection (Uvnäs-Moberg et al., 2005; Welch and Ludwig, 2017). In the following section, we will explore studies of maternal tactile stimulation and/or OT administration in the postnatal period and their potential influence on offspring state regulation and biology as well as the offspring own parenting behaviors.

## Rodent Maternal Tactile Stimulation and Offspring Developmental Outcomes in Relation to OT

OT has long been known to be implicated in basic female reproductive events, such as birth (Dale, 1906, 1909) and breastfeeding (Ott and Scott, 1910). That OT could have a role in modulating social behavior during the transition to maternal behavior in the goat was first proposed by Klopfer and Klopfer (1968) and Klopfer (1971). Pedersen and Prange (1979) and Pedersen et al. (1982) tested that hypothesis by intercerebroventricular injections of OT in virgin rats, which, in an estrogen-dependent manner, stimulated the onset of full maternal behavior.

Research in the behavioral, neurological, and epigenetic outcomes of maternal tactile stimulation has been made possible through the serendipitous observation that rodent mothers within the same species will naturally vary in the amounts of contact, licking and grooming (LG), and arched back nursing (ABN) they direct at their pups during lactation, with significant positive correlations to adult blood pressure (Myers et al., 1989a,b).

To test the developmental and potential non-genomic outcomes of these natural variations in maternal caregiving, Francis et al. (1999) employed cross-fostering as well as an environmental manipulation imposed during early development, which alters maternal behavior, especially maternal LG-ABN of pups. They were able to convincingly demonstrate that variations LG-ABN serve as the basis for a non-genomic transmission of both maternal behavior and stress reactivity.

A major breakthrough in demonstrating how OT systems are linked to the specific maternal behavioral component of pup-directed licking and grooming and arched back nursing was achieved with subsequent studies showing that lactating females characterized as Low LG-ABN during the first week postpartum have decreased levels of OTR binding in the medial preoptic area of the hypothalamus compared to high LG-ABN dams (Champagne et al., 2001; Francis et al., 2000, 2002), as do their offspring. Furthermore, a reduction in pup-directed LG-ABN among high LG-ABN dams can be achieved through central infusion of a selective OTA, in contrast to insignificant effects in low LG-ABN dams (Champagne et al., 2001). Pedersen and Boccia (2002) replicated this finding and also observed an increase in maternal self-grooming, following infusion of an OTA, leading to the conclusion that one of the functions of OT during lactation may be to shift maternal oral grooming away from themselves and instead direct it at the offspring. The expression of estrogen receptor in the medial preoptic area has been shown to be implicated in the expression of OTR and is furthermore susceptible to caregiving environments, as demonstrated through cross-fostering studies (Champagne et al., 2006). As for developmental outcomes, the level of maternal LG-ABN has been found to correlate positively with offspring maternal behavior (specifically LG-ABN behavior) during adulthood, behavioral and endocrine responsiveness to stress, emotionality, performance in tests of spatial learning and object recognition, and glucocorticoid receptor genetic expression in the hippocampus (Liu et al., 1997, 2000; Francis et al., 1999; Caldji et al., 2000).

Pedersen and Boccia (2002) have provided longitudinal evidence that maternal care regulates OT activity in pups, which in turn influences the development of OTR and the development of maternal care when the experimental animals become mothers themselves. Oxytocin receptor concentrations in areas of the adult brain where oxytocin stimulates maternal behavior or diminishes anxiety and adrenal axis responses to acute stress have been shown to be positively related to LG-ABN received during infancy. In a separate study on postnatal days 2–10, pups (F1) were treated with either subcutaneous administration of OT, an OTA, or saline vehicle. At 3–4 months of age, the F1 dams were mated and LG-ABN frequency measured during PND 2 and 5. Through videotaping the maternal behavior of the F0 rearing dams, the outcomes were controlled for variability in maternal caregiving received during the infancy of the F1 dams. Compared to F1 dams treated with saline vehicle, LG-ABN frequency was significantly higher in F1 dams that received postnatal OT and significantly lower in F1 dams treated with OTA (Pedersen and Boccia, 2002).

Furthermore, attesting to the long-term effects of early OT exposure, rats given subcutaneous OT injections or exposed to brief stroking episodes on the ventral side of the abdomen once a day in the postnatal period result in adult decreased blood pressure (Holst et al., 2002) and corticosterone levels (Sohlström et al., 2000) as well as increased adult nociceptive thresholds and increased functioning of central alpha-2-adrenoreceptors (Díaz-Cabiale et al., 2004), the latter with implications for improved stress regulation (Uvnäs-Moberg et al., 2014).

Early studies of the development of OTR expression in rodents indicate that they arise in several brain sites at PND 5 and then gradually increase in density until PND 10–14, to then disappear in most sites at the time of weaning (Shapiro and Insel, 1989; Tribollet et al., 1991). The adult pattern of OTR expression begins to emerge during puberty, which is the other highly plastic period (Gimpl and Fahrenholz, 2001). One of the few brain structures in which oxytocin receptors appear during the early phase and then persist into adulthood is the amygdala (Pedersen and Boccia, 2002). This early expression of OTR in central brain regions coincides with the period where maternal LG-ABN exerts its strongest effects on stress responses and maternal behavior (Meaney et al., 1996). Confirming the existence of this sensitive period, studies employing an environmental manipulation known to increase maternal pup-directed licking and grooming (handling, see Meaney et al., 1996; Lee and Williams, 1974) demonstrate that such manipulation is effective at attenuating stress responses if experienced during the first postnatal week but less so or not at all effective if the handling is experienced exclusively during the second and third postnatal weeks, respectively (Levine and Lewis, 1959; Meaney and Aitken, 1985). Providing an even more fine-grained analysis of the developmental timing of the effects of maternal care on the offspring’s maternal sensitivity, cross-fostering high LG-ABN and low LG-ABN pups at PND 6 produces a shift in maternal sensitivity when the experimental pups become dams themselves so as to correspond to the rearing conditions of the adoptive dam. However, cross-fostering at PND 10 does not demonstrate an effect on maternal sensitivity (Peña et al., 2013).

Through fine-grained behavioral analyses of three main maternal behaviors, which constitute the LG-ABN complex (licking and grooming; simple physical contact lasting 30 s or more; skin-to-skin contact involving large areas of the pup’s body lasting 1 m or more) directed at individual 14 PND pups, Kojima et al. (2012) have shown that, of these three main behaviors, only skin-to-skin contact correlated with hypothalamus OT, but not with peripherally measured plasma OT. Interestingly, it is the thermotactile component of skin-to-skin contact—i.e., the mother reliably providing cutaneous warmth—which appears to potentiate pup OT concentrations as well as facilitate their acquisition of a filial huddling preference. Neither suckling, milk reward, maternal licking, nor mechanotactile stimulation such as stroking appear to affect the acquisition of a filial huddling preference at that particular stage of development (approx. PND 14) (Alberts and May, 1984; Kojima and Alberts, 2009, 2011; Kojima et al., 2012).

These findings, however, do not preclude that the specific licking and grooming behavioral component may influence emerging OT systems during PND 1–13, owing to the extremely dynamic development of OT systems and the large changes in levels and types of maternal care during the preweaning period. In fact, partial reversal of the maternal caregiving deficits normally associated with being raised without a mother was achieved through experimenter-provided licking and grooming-like stimuli during PND 4–20 only (Gonzalez et al., 2001; Lovic and Fleming, 2004).

From these combined findings, we can conclude that rodent pup OT systems are amenable to maternal sensory stimulation, notably licking and grooming and skin-to-skin contact and its concomitant provision of cutaneous warmth. The early shaping of OT systems appears to predict both the offspring’s own maternal behavior as well as epigenetic, endocrine, cognitive, and stress-regulatory outcomes.

## Extended Parent–Infant Physical Contact

We now turn to our analysis of a potential candidate for the clinical application of the extant findings of the OT research summarized above—extended parent–infant physical contact (EPIC), as described in section “Introduction.” We will address the following questions: (1) Does EPIC involve the human equivalents of the specific maternal caregiving behaviors found to influence OT systems in rodents? (2) Has EPIC been found to influence OT levels in parents and full-term infants? (3) Does EPIC positively influence those particular outcome domains that have been documented following exogenous release of OT or externally administered OT? (4) Does the support of universal EPIC among caregivers of healthy full-term infants constitute a clinically relevant and scalable intervention?

### Does EPIC Involve the Human Equivalents of the Specific Parental Caregiving Behaviors Found to Influence OT Systems in Rodents?

Whereas caution should be exerted in translating findings from animal research to humans (Perel et al., 2007), the extant findings from both animal and human research on the links between non-noxious tactile or other somatosensory stimulation and an increase in central or peripheral OT levels suggest that similar mechanisms may be at play (Stock and Uvnäs-Moberg, 1988; Matthiesen et al., 2001; Morhenn et al., 2012). So far, we have evidence from animal studies that maternal pup-directed licking and grooming as well as maternal ventral skin-to-skin contact lasting more than 1 min per bout influence offspring OT systems (Francis et al., 2000; Champagne et al., 2001; Kojima et al., 2012).

We begin our analysis with examining parallels of extended bouts of rodent skin-to-skin contact to large parts of the pups’ body (Kojima et al., 2012) with the specific somatosensory stimulation associated with EPIC. As briefly described in section “Introduction,” EPIC covers a range of relatively similar practices, which all have in common that the infant is placed ventrally toward the caregiver for prolonged periods, usually lasting significantly longer than 1 min per bout of carrying (Anisfeld et al., 1990; Pisacane et al., 2012; Williams and Turner, 2020). Such ventral–ventral contact exerts a static pressure and is likely to activate specific C-fibers, which innervate the breasts and the chest and which are connected to the vagal nuclei, which are possibly in functional contact with cells producing OT in the hypothalamus (Eriksson et al., 1996; Nelson et al., 1998). However, not only non-myelinated C-fibers are activated, but myelinated sensory nerve fibers also play a role in inducing antistress effects, in which OT is likely to play a role (Sato and Schmidt, 1987). Indeed, ventral–ventral skin-to-skin contact have been shown to increase both parental and infant salivary OT levels and to decrease salivary cortisol levels, inducing less anxiety and more synchronous and responsive in parents (Cong et al., 2015; Vittner et al., 2018).

Furthermore, similar to the mechanisms uncovered in rodents (Alberts and May, 1984; Kojima and Alberts, 2009, 2011; Kojima et al., 2012; Meyer and Alberts, 2016), maternal provision of cutaneous warmth during ventral–ventral contract activates cutaneous nerves in the infant, leading to release of OT in the infant, which decreases sympathetic nervous system activity, which in turn reduce infant vasoconstriction, resulting in higher infant skin temperature (Bystrova et al., 2003, 2007; Moberg et al., 2020). Detailed analyses of maternal chest temperature following birth demonstrate both a higher temperature than that of the infant as well as higher variability in a pulsatile pattern when in skin-to-skin contact. This pulsatile pattern is highly adapted to stimulate the increase in infant temperature, as a varying temperature activates the infant cutaneous sensory nerves more effectively than a stable temperature, leading to a synchronization of mother and infant temperatures (Bystrova et al., 2003).

As for the specific mechanotactile maternal behavior of rodent licking and grooming, a human equivalent is considered to be maternal gentle stroking of the infant, at the speed and pressure level, which has been shown to activate a class of touch sensitive nerve fibers named C-Tactile afferents, inducing epigenetic and stress-regulatory effects similar to those found in rodent licking and grooming studies (Murgatroyd et al., 2015; Sharp et al., 2015; Pickles et al., 2017). Furthermore, the effects of this type of affectionate touch has been shown to mirror more generally those that have been reported following endogenous release and exogenous administration of oxytocin and are likely partially mediated by C-Tactile afferents, as reviewed by Walker et al. (2017). Although this is so far not quantitively examined, casual observations and video documentation (Bigelow and Gillis, 2010) of parents engaging in EPIC demonstrate a proclivity of parents to also perform—concurrently—infant-directed affectionate touch in the form of light stroking.

In conclusion, EPIC involves the human functional equivalents of the specific somatosensory stimuli that have been shown in rodent research to influence offspring OT systems, especially ventral–ventral skin-to-skin contact, providing cutaneous warmth, and may also include parental gentle stroking, considered the functional equivalent of rodent licking and grooming. There is still a large research effort ahead to first of all investigate the many potential configurations of combinations of concurrent ventral contact and maternal stroking among human caregivers, and second, to tease out the relative contributions of these two discrete caregiving behaviors to the development of infant OT systems during the period of the infant life span where EPIC is normally conducted.

### Has EPIC Been Found to Influence OT Levels in Parents and Full-Term Infants?

So far, no studies have been conducted on full-term infants and their caregivers. However, Vittner et al. (2018), employing the methodology developed by Sue Carter et al. (2007), measured salivary OT and cortisol levels in stable preterm infants and their mothers and fathers before, during, and after a session of skin-to-skin contact in the neonatal intensive care unit (NICU) environment. Salivary OT levels increased significantly during SSC for mothers, fathers, and infants. Cong et al. (2015) similarly measured parental salivary OT before, during, and after a single session of SSC and found both maternal and paternal OT levels to be significantly increased during SSC from baseline. Paternal OT continued to be maintained at a higher level after the SSC session, whereas maternal OT declined.

The question of whether EPIC influences OT levels is, however, complicated by the possibility that much of OT’s effects are exerted through active fragments, which, in the case of skin-to-skin contact, exert antistress effects by acting on alpha-2-adrenoreceptors, decreasing sympathetic nervous system activity. Furthermore, in contrast to maternal predominant brain-located OT response to infant suckling, skin-to-skin contact may to a greater degree activate OT production in peripheral systems, such as the blood vessels and the gastrointestinal tract (Uvnäs Moberg et al., 2019).

### Does EPIC Positively Influence the Main Outcome Domains That Have Been Documented Following Endogenous Release and Externally Administered OT?

From the literature cited in the above, three different main domains of outcomes can be defined: socioemotional, stress regulation, and maternal caregiving behavior. If EPIC is to qualify as an evidence-based clinically relevant universal intervention aimed at optimizing healthy full-term infants’ developing OT systems, studies should support the effects of EPIC in these domains.

#### EPIC and Socioemotional Outcomes

Socioemotional outcomes, especially the capacity to engage in trustful and mutually rewarding relations as measured via, e.g., attachment security, are critical, considering the accumulation of evidence regarding the fundamental role of attachment in an extensive range of developmental outcomes (Garner et al., 2012; Thompson, 2016; Ehrlich and Cassidy, 2019). Hence, in the following, a relatively in-depth description of EPIC-related outcomes is provided.

The first controlled study of EPIC randomized low socioeconomic status (SES) status US mothers after the birth of their full-term healthy infants to receive either a plastic seat (*n* = 26) or a soft cloth structured infant carrier (*n* = 23), designed to carry the infant in ventral–ventral contact (Anisfeld et al., 1990). The participants were encouraged to make daily use of the products provided. To document actual usage, pedometers were sewn inside the soft cloth infant carriers. At 13 months of age, the strange situation protocol was administered. The strange situation is considered the gold standard for measuring infant attachment quality, dividing infants into categories of either secure or insecure attachment, with implications for the effectiveness of the infants’ use of the caregiver as a secure base for exploration of the environment and as stress regulator when the infant faces threatening or ambiguous environments (Ainsworth et al., 1978; Main et al., 1985). In the plastic seat control group, 38% of the infants were classified as secure, in contrast to 83% in the infant carrying group. Among the mothers supplied with infant carriers, the subgroup categorized as high users (through the pedometer readings) had 93% securely attached infants, in contrast to 57% in the subgroup of low/moderate users, suggesting a dose–response relation between infant carrying and positive infant attachment outcomes.

In a recent partial replication, targeting another high-risk population, Williams and Turner (2020) randomized US teenage mothers to either receive an infant carrier (*n* = 16, min. 1 h/day of infant carrying for 3 months) or high contrast baby books (*n* = 17) at 2–4 weeks postpartum. At 7 months of age, infant behaviors during the Still-Face Paradigm (Tronick et al., 1978) were coded through an algorithm derived from the Infant Global Rating Scales to determine attachment type (Murray et al., 1996; Abbott, 2016). Infants of mothers issued an infant carrier were more likely to have secure attachments and less likely to have disorganized attachments compared to infants whose mothers were issued baby books. Hours spent in an infant carrier were positively correlated with secure attachment and negatively correlated with disorganized attachment.

The effects of increasing maternal provisions of SSC during the first postpartum month has also been investigated in a Canadian study of low-risk middleclass mothers (Bigelow and Power, 2012). The experimental group (*n* = 28) was asked to engage in daily SSC and consequently held their infants for 5 h/day during the first postpartum week and 3 h/day during postpartum weeks 2–4. A control group (*n* = 52) was established for whom no recommendations for SSC were issued, and consequentially, very little SSC was provided. At 1 week and 1, 2, and 3 months, the Still-Face Paradigm (Tronick et al., 1978) was employed to track the infants’ socioemotional development. The SSC infants were found to have an accelerated socioemotional development across these measurements, both in comparison to the control group and to normative development, found in other samples. Furthermore, at the age of 3 months, the SSC infants were found to increase their non-distress vocalizations during the still-face episode compared to the interactive phases of the Still-Face Paradigm, demonstrating the SSC infants’ bids to reengage the unresponsive mother. At that age, such unresponsive maternal behavior will usually frustrate and weaken infant social bidding. A meta-analysis suggests a link between such more eliciting behavior and positive affect during the still-face episode and infant secure attachment at 12 months (Mesman et al., 2009).

A follow-up study at child age 9 years had these Canadian children and their mothers engage in conversation about remembered emotional events in the children’s lives. The conversations were assessed on the Autobiographical Emotional Events Dialogue. Those mother–child dyads, which has engaged in SSC during early infancy, demonstrated more engagement and reciprocity and had a greater likeliness of being classified as emotionally matched. These findings support that early provisions of skin-to-skin contact, which is likely to have influenced emerging OT systems, may have set the mother–child dyads on a mutually socially positive developmental course (Bigelow et al., 2018).

Corroborating the contributions of the thermotactile component of EPIC—the reliable maternal provision of cutaneous warmth—to infant OT systems and socioemotional development, SSC provided postpartum increased neonate peripheral temperatures, relative to control neonates, which were either separated or held in arms while dressed (Bystrova et al., 2003). Furthermore, the neonates’ capacity to maintain high peripheral temperature through their exposure to maternal cutaneous warmth during the first postpartum days predicted their significantly improved socioemotional competencies, relative to control infants, 1 year after birth, revealing a remarkable continuation of optimum physiological regulation to the domain of psychological regulation (Bystrova et al., 2009).

#### Stress Regulation

There are no studies examining the effects of EPIC in full-term infants on the classical indices of stress regulation, such as cortisol reactivity. However, there are several studies that provide indirect evidence of an improved infant stress regulation through displays of more optimum state control as well as physiological and behavioral calming. For example, when carried in the arms of a briskly walking mother, infants aged < 6 months demonstrated reduced crying, body movement, and heart rate, as well as increased heart rate variability [the latter indicative of a more optimum autonomic nervous system regulation (Porges et al., 2019)], compared to stationary holding or being placed in a cot (Esposito et al., 2013).

Similarly, 2-day-old healthy full-term infants placed separately to their mother in a cot and exposed to maternal SSC for 1 h in each location demonstrated an 82% decrease in quiet sleep during maternal separation in comparison to SSC, as well as a significantly greater latency to enter into quiet sleep, indicative of activation of the sympathetic nervous system (Morgan et al., 2011). Whereas there are no longitudinal data for the long-term effects on sleep organization of early SSC provision among full-term infants, a longitudinal study of premature infants exposed to maternal SSC for at least 14 days of 2 h/day during their NICU stay demonstrated significantly more optimum sleep organization, with more quiet sleep and quiet alert states at term (Feldman et al., 2002). Follow-up at child age 10 years showed that the children exposed to SSC during their prematurity had a significantly greater sleep efficiency and significantly shorter bouts of nighttime awakenings, as measured through five nights, wearing an actigraph, in comparison to the children who received standard incubator care during prematurity (Feldman et al., 2014). Such findings of long-term effects of an early touch-related intervention, which presumably influences developing OT systems, are consistent with the rodent OT research where subcutaneous OT injections or exposure to brief stroking episodes on the ventral side of the abdomen once a day in the postnatal period results in adult decreased blood pressure (Holst et al., 2002), corticosterone levels (Sohlström et al., 2000), increased adult nociceptive thresholds, and increased functioning of central alpha-2-adrenoreceptors (Díaz-Cabiale et al., 2004), the latter positively influencing stress regulation (Uvnäs-Moberg et al., 2014). Nevertheless, to fully corroborate the existence of similar mechanisms for such strong early programming effects in the more developed and physiologically stable full-term infant, longitudinal studies targeting full-term infants and their parents are required.

A study of differences between core and peripheral temperatures across the first two days of life of healthy infants born at term demonstrated how being in SSC with the mother ensures a positive neonate heat balance, whereas being placed separately in the cot results in heat loss of up to 70 W/m^2^, which is close to what a neonate can compensate for (Fransson et al., 2005). Hence, during the first few days of life, SSC is effective in avoiding cold stress (Bystrova et al., 2003).

Infant crying is an indicator of stress, hence less crying ought to suggest an improved stress regulation. EPIC has been shown to reduce infant crying in two trials with healthy full-term infants (Hunziker and Barr, 1986; St James-Roberts et al., 2006), whereas EPIC may not be effective in alleviating colic (distress > 3 h/day) (Barr et al., 1991; St James-Roberts et al., 1995, 2006), suggesting that colic/unsoothable crying may have as yet understood underlying neurological causes (St James-Roberts, 2008).

#### Maternal Caregiving

Of the three studies that have investigated EPIC effects on infant social development two have also studied potential effects on maternal caregiving quality. When the infants in Anisfeld et al. (1990) study had reached the age of 3.5 months, 15 min film video recordings of each mother–infant dyad were made of a free-play session. Those mothers who had been issued an infant soft structured carrier at birth were found to be more contingently vocally responsive and showed a trend toward being more sensitive toward their infant’s cues, state, and rhythm, as measured by the sensitivity-related scale from Crnic’s system (Crnic et al., 1983), in comparison to the control group, which was issued a plastic seat. According to a logistic regression analysis, the differences in attachment outcomes were not solely due to differences in maternal responsiveness. Hence, infant carrying appeared to have an effect beyond that attributable to maternal responsiveness.

In their study of 80 middle-class mother–infant dyads, Bigelow et al. (2014) employed the Nursing Child Assessment Feeding Scale (NCAFS), which measure among others maternal sensitivity to infant cues well as responsivity to infant distress at each visit at 1 week and 1, 2, and 3 months postpartum. They, however, found no significant differences in mother–infant interactions between SSC mothers and controls.

Considering that both of the above studies found significant differences in infant socioemotional outcomes, it would appear that maternal sensitivity-oriented measures do not predict infant outcomes well. This is in accordance with meta−analytic data, showing that sensitivity as originally defined by Ainsworth et al. (1978) accounts for a low percentage of the variance in attachment—approximately 6% and only approximately 2% among families with low socioeconomic status (Wolff and Van IJzendoorn, 1997). Relevant to our interest in physical contact and OT, Woodhouse et al. (2020) recently demonstrated which of the many maternal behaviors measured via the sensitivity construct have the strongest impact on infant attachment outcomes. They found that among low SES mothers, it was their capacity to engage in ventral–ventral contact in order to soothe their distressed infants to a fully calm and regulated state at least 50% of the time as well as allow relatively uninterrupted exploration, which mattered the most. Mastering these specific maternal behaviors increased their infants’ chances of developing a secure attachment from about 30 to 71%. These findings mirror those of the early attachment pioneers, which also noted strong links between maternal incompetence or rejection of providing physical contact and infant attachment quality (Ainsworth et al., 1978; Main and Stadtman, 1981).

One of the central findings of the rodent maternal developmental programming was the non-genomic transmission of maternal caregiving behaviors (Champagne and Meaney, 2001). Currently, no longitudinal cross-generational human data exist for the maternal capacity to engage in extended or even brief but sufficiently long bouts of ventral–ventral physical contact (cf. Woodhouse et al., 2020). However, during the home observations that was part of the pioneering work of Ainsworth et al. (1978) conducted to develop the Strange Situation for the assessment of infant attachment quality, some mothers were found to display aversion to physical contact stably across the first year of their childrens’ lives, with resultant infant aggression, conflict behavior, and ultimately insecure-avoidant attachment (Main and Stadtman, 1981). Presumably, such mothers would be the human equivalent of the low LG-ABN rodent dams (Champagne and Meaney, 2001). Employing the Adult Attachment Interview, Main (1990) found links between maternal unresolved representations of received rejections to their childhood bids for physical contact toward their mother and contemporaneous observations of the interviewed mothers’ aversion to physical contact with their infants. Hence, we have some retrospective longitudinal human evidence for the “like mother like daughter” mechanism found in rodents (Champagne and Meaney, 2001).

#### Does the Support of Universal EPIC Among Caregivers of Healthy Full-Term Infants Constitute a Clinically Relevant and Scalable Intervention?

For any intervention to be considered evidence-based in modern medicine, it would have to live up to the requirements of being (1) sufficiently empirically supported, (2) in accordance with the individual practitioner’s clinical judgment, and (3) acceptable to the client. Whether an intervention is thought to be sufficiently empirically supported to warrant implementation presumably rests on several factors: the urgency of the medical challenge presented, efficacy and feasibility of alternative interventions, the relative financial and organizational demands of all potential interventions.

Do human suboptimum OT systems constitute a medical challenge that requires urgent action? First of all, how to meaningfully quantify proportions of the general population, affected by suboptimum OT systems? Links between OT, maternal attachment representations, maternal caregiving behavior, and infant attachment outcomes (Strathearn et al., 2009; Shah et al., 2010; Atzil et al., 2011; Galbally et al., 2011) suggest that population estimates of infant and adult insecure attachment might serve as a meaningful proxy. On this basis, approximately 40% of the general population will suffer from suboptimum OT systems/insecure attachment (van Ijzendoorn and Sagi-Schwartz, 2008; Bakermans-Kranenburg and van IJzendoorn, 2009; Moullin et al., 2014). Insecure attachment/suboptimum social functioning is increasingly recognized for its role in human physical and psychological disease (Puig et al., 2013; Holt-Lunstad, 2018; McDade and Harris, 2018; Ehrlich and Cassidy, 2019) as is the prophylaxis of the capacity to have positive social connections in the face of childhood trauma (Garner et al., 2012). This ought to make the prevention of children developing suboptimum OT systems an urgent challenge.

Meta-analysis of current infant attachment interventions, which tend to have a maternal representational (e.g., Juffer et al., 2017; Juffer and Bakermans-Kranenburg, 2018) and/or behavioral focus (Bakermans-Kranenburg et al., 2003; Dozier and Bernard, 2017; Caron et al., 2018; Hoye and Dozier, 2018), reveal relatively modest outcomes for infant attachment (Bakermans-Kranenburg et al., 2003; Wright and Edginton, 2016). Furthermore, the interventions mostly require relatively long and frequent individual therapeutic sessions, making them costly to implement (Wright and Edginton, 2016).

Judging from the studies conducted this far, supporting both low- and high-risk parents to successfully engage in EPIC appears feasible (Anisfeld et al., 1990; Bigelow and Power, 2012; Williams and Turner, 2020). However, postpartum specialized clinical support of EPIC may be necessary for greater compliance. To give an illustration of this: An Italian study provided 200 middleclass mothers either an infant carrier after the delivery (of which 65% were cesarean) of their full-term healthy infants or a breastfeeding instruction pamphlet. Of the 100 mothers provided a carrier, 69 utilized it for at least 1 h/day during the first month of life, while 31 did not use it at all. Of the non-using mothers, 15 ascribed their non-use to cesarean pain. Other reasons for non-use was not feeling comfortable with the carrier or the infant crying while in the carrier (Pisacane et al., 2012). Theoretically, based on the findings of Main (1990) described above, some parents may have challenges in engaging in EPIC, based on their own childhood received rejections of their bids for physical contact. Parents’ capacity to engage in EPIC may also be influenced by their own unresolved childhood trauma and autistic traits (Voos et al., 2013; Iyengar et al., 2014, 2019; Peled-Avron and Shamay-Tsoory, 2017). At this early stage of development of clinical implementation of EPIC, very little information exists as to the proportion of parents likely to face challenges engaging in close physical contact with their infant. Consequentially, therapeutic approaches to resolve such challenges have also yet to be defined and confirmed through testing. Potential therapeutic pathways could involve tactile components, such as massage, body psychotherapy, or psychomotor therapy, capitalizing on the OT research, which demonstrates links between received tactile stimulation, OT levels, and subsequent willingness and capacity to purposefully engage in conspecific social contact (Stock and Uvnäs-Moberg, 1988; Uvnäs-Moberg et al., 2005; Lukas et al., 2011). Massage and other manual touch-related therapies, such as reflexology, have been shown to increase salivary and plasma OT levels in male (Morhenn et al., 2012; Li et al., 2018), female (Morhenn et al., 2012), puerperal (Yokoyama et al., 1994; Matthiesen et al., 2001), and autism spectrum disorder subjects (Tsuji et al., 2015), reduce stress and anxiety in a psychiatric inpatient ward (Garner et al., 2008), and, when delivered in several sessions antenatally, reduce labor pain and/or labor duration in parturient women (Nabb et al., 2006; Dolatian et al., 2011; McCullough et al., 2017), indicative of improved OT functioning (Uvnäs-Moberg et al., 2019). Other potential therapies, with a rich history of clinical application and presumably acting on and via OT mechanisms, could include parent–infant attachment-promoting body psychotherapy (Harms, 2015) or psychomotor interventions (Emck and Scheffers, 2019). However, all of these abovementioned therapies are yet to be rigorously and formally evaluated for the effectiveness in resolving parental discomfort or incapacity in relation to extended parent–infant contact, attesting to the scant research efforts invested toward implementation challenges of parent–full-term infant contacts practices in clinical settings, in stark contrast to the current state for parental–preterm infant contact (kangaroo mother care) (Bergh et al., 2016; Chan et al., 2017; Smith et al., 2017; Nimbalkar and Sadhwani, 2019).

## Conclusion

Impressive progress has been made in the past decades in understanding the OT-related biology of somatosensory stimulation and its links to animal and human social interaction, especially parent–offspring behavior (Champagne et al., 2001; Uvnäs-Moberg, 2015; Insel, 2016; Feldman and Bakermans-Kranenburg, 2017). Much of the research in potential clinical applications of these biological discoveries have been invested in pharmacological approaches, targeting various social deficits, albeit with limited success (Bakermans-Kranenburg et al., 2013; Grimm et al., 2014; Hofmann et al., 2016; Quintana and Woolley, 2016). In recent years, a shift from the focus on primarily negative aspects of maternal mental health, such as symptoms of depression, anxiety, or states of distress and child maladaptation to positive maternal health and child resilience-inducing processes signals a growing awareness of the vital necessity to move at least some of the available research resources toward more preventative approaches (Feldman, 2020; Phua et al., 2020). Similarly, the emerging evidence for links between the capacity to engage in trustful relations—being securely attached—and physical health (Puig et al., 2013; Holt-Lunstad, 2018; McDade and Harris, 2018; Ehrlich and Cassidy, 2019) as well as the large proportion of the general population showing deficits in that very capacity (van Ijzendoorn and Sagi-Schwartz, 2008; Bakermans-Kranenburg and van IJzendoorn, 2009; Moullin et al., 2014) gives even greater urgency to confirm and implement scalable interventions. Supporting parents to engage in extended physical contact with their full-term healthy infants appears to bring about many of the animal OT- and sensory-stimulation-related effects and furthermore may be a comparatively low-cost and thus scalable intervention (Williams and Turner, 2020). With these perspectives, a clinical application of the hard-won insights over the past decades into fundamental affiliative and resilience-inducing biological mechanisms may be within reach.

## Author Contributions

HN wrote the manuscript.

## Conflict of Interest

The authors declare that the research was conducted in the absence of any commercial or financial relationships that could be construed as a potential conflict of interest.

## Abbreviations

EPIC: extended parent infant contact
LG-ABN: licking and grooming and arched back nursing
OT: oxytocin
OTA: oxytocin antagonist
OTR: oxytocin receptors
PND: postnatal days
SSC: skin-to-skin contact
VP: vasopressin.
